# Remote cochlear implant programming: a systematic review of clinical effectiveness and implementation outcomes

**DOI:** 10.1007/s00405-026-10068-4

**Published:** 2026-03-05

**Authors:** Muhammed Ayas, Jameel Muzaffar, Marwa Madi, Ahmad AlAmadi, Mira Koleva, Manohar L. Bance

**Affiliations:** 1 Audiology & Speech Language Pathology, College of Health Sciences, University of Sharjah, Sharjah, United Arab Emirates; 2 Department of Clinical Neurosciences, University of Cambridge, Cambridge, UK; 3 Department of ENT, University Hospitals Birmingham NHS Foundation Trust, Birmingham, UK; 4 Department of ENT, Cambridge University Hospitals NHS Foundation Trust, Cambridge, UK; 5 Cambridge Hearing Group, University of Cambridge, Cambridge, UK; 6 Emmeline Centre, Cambridge University Hospitals NHS Foundation Trust, Cambridge, UK; 7 Department of Applied Health Sciences, University of Birmingham, Birmingham, UK; 8 Sharjah City for Humanitarian Services, Sharjah, United Arab Emirates; 9 Advanced Hearing and Balance centre, Dubai, United Arab Emirates

**Keywords:** Remote programming, Cochlear implants, Tele-audiology, Speech perception, Implementation outcomes

## Abstract

**Purpose:**

Remote cochlear implant (CI) programming is increasingly used to expand access to follow-up care. This systematic review evaluated the clinical effectiveness and implementation outcomes of synchronous, asynchronous and hybrid remote CI programming in adults and children.

**Methods:**

PubMed, Embase, Scopus, CINAHL and the Cochrane Library were searched from January 2010 to July 2025. Eligible studies were randomised or observational designs reporting clinical outcomes (speech perception, aided thresholds, impedances, threshold/comfort levels) and/or implementation outcomes (feasibility, satisfaction, technical performance). Two reviewers independently screened, extracted data and assessed risk of bias. Given heterogeneity in design and outcomes, a narrative synthesis was conducted.

**Results:**

Eleven studies (*n* = 356 CI recipients) met inclusion criteria. Across comparator studies, remote programming produced no clinically meaningful differences relative to in-person programming for speech perception (7/7 studies) or aided thresholds (5/5), and programming parameters remained stable (5/5). Feasibility was high, with session-completion rates typically ≥ 85%. CI recipients and clinicians reported favourable satisfaction, citing reduced travel burden and increased flexibility. Key challenges included intermittent connectivity, need for additional preparation or troubleshooting, limited digital literacy among older adults, and caregiver dependency in paediatric sessions.

**Conclusion:**

Remote CI programming yields clinical outcomes comparable to in-person programming and is feasible and well accepted across delivery models. However, small sample sizes, limited paediatric evidence, inconsistent technical reporting and a lack of cost-effectiveness data limit confidence in large-scale adoption. Standardised technical endpoints and rigorous multicentre evaluations are required to support scalable and equitable implementation.

## Introduction

Cochlear implants (CI) are a well-established intervention for individuals with moderate to profound sensorineural hearing loss who receive limited benefit from hearing aids. Programming, or “mapping,” is an essential component of post-operative care and involves periodic adjustment of device parameters to optimise auditory performance. These appointments are usually conducted in specialised centres by trained audiologists or CI specialists. However, access to such centres is frequently constrained by geographic distance, socioeconomic barriers and mobility limitations, particularly among rural residents, children and older adults. Rural CI recipients are significantly more likely to delay or miss follow-up appointments due to travel burden and logistical challenges [1, 2] and shortages of CI-trained audiologists further limit timely and equitable access to care in many regions [3].

Telehealth-enabled remote CI programming has emerged as a promising alternative, Remote CI programming enables clinicians to adjust stimulation parameters and perform diagnostics without requiring in-person attendance. Remote CI programming can be delivered through synchronous models (real-time videoconferencing), asynchronous models (offline data upload and clinician review) or hybrid approaches combining remote and in-clinic components [4, 5]. These models are now supported across major CI manufacturers (Advanced Bionics, Cochlear and MED-EL) and are increasingly incorporated into routine clinical pathways.

Early studies suggest that remote CI programming can yield outcomes comparable to traditional in-person mapping for parameters such as aided thresholds, device integrity and subjective sound quality [5, 6]. However, variability in testing environments, connectivity demands and user interaction may influence programming accuracy and safety. A recent meta-analysis reported no significant differences in speech-perception outcomes [7], but no review has compared synchronous, asynchronous and hybrid models in relation to objective CI metrics such as threshold(T)/Comfort(C) level and impedances as well as technical reliability. Tele-audiology literature has examined remote hearing care more broadly [8, 9], yet it has not synthesised clinical and implementation outcomes specific to CI programming, nor compared synchronous, asynchronous and hybrid models. Recent work on remote-care outcome sets remains descriptive and does not appraise effectiveness or feasibility across paediatric and adult CI users [10].

Therefore, this systematic review addresses the following question: What are the clinical effectiveness outcomes and the implementation outcomes of remote CI programming in paediatric and adult populations? To answer this question, we synthesised evidence across multiple outcome domains, including speech perception, aided thresholds, impedances and threshold/comfort levels, as well as feasibility, satisfaction, technical reliability and caregiver involvement. By grounding the synthesis in clinically relevant CI measures, this review aims to clarify the conditions under which remote programming can provide reliable, safe and scalable follow-up care.

## Materials and methods

This review assessed the effectiveness and implementation of remote cochlear implant (CI) programming across synchronous, asynchronous and hybrid delivery models. The review followed the Preferred Reporting Items for Systematic Reviews and Meta-Analyses (PRISMA) guidelines [11]. A comprehensive search of the literature was conducted in MEDLINE (PubMed), Embase, Scopus, CINAHL and the Cochrane Library, covering studies published between January 2010 and July 2025. Search terms included medical subject headings and keywords related to cochlear implants, telehealth, tele-audiology, remote programming and remote fitting, combined using Boolean operators to refine the results.

**Fig. 1:**
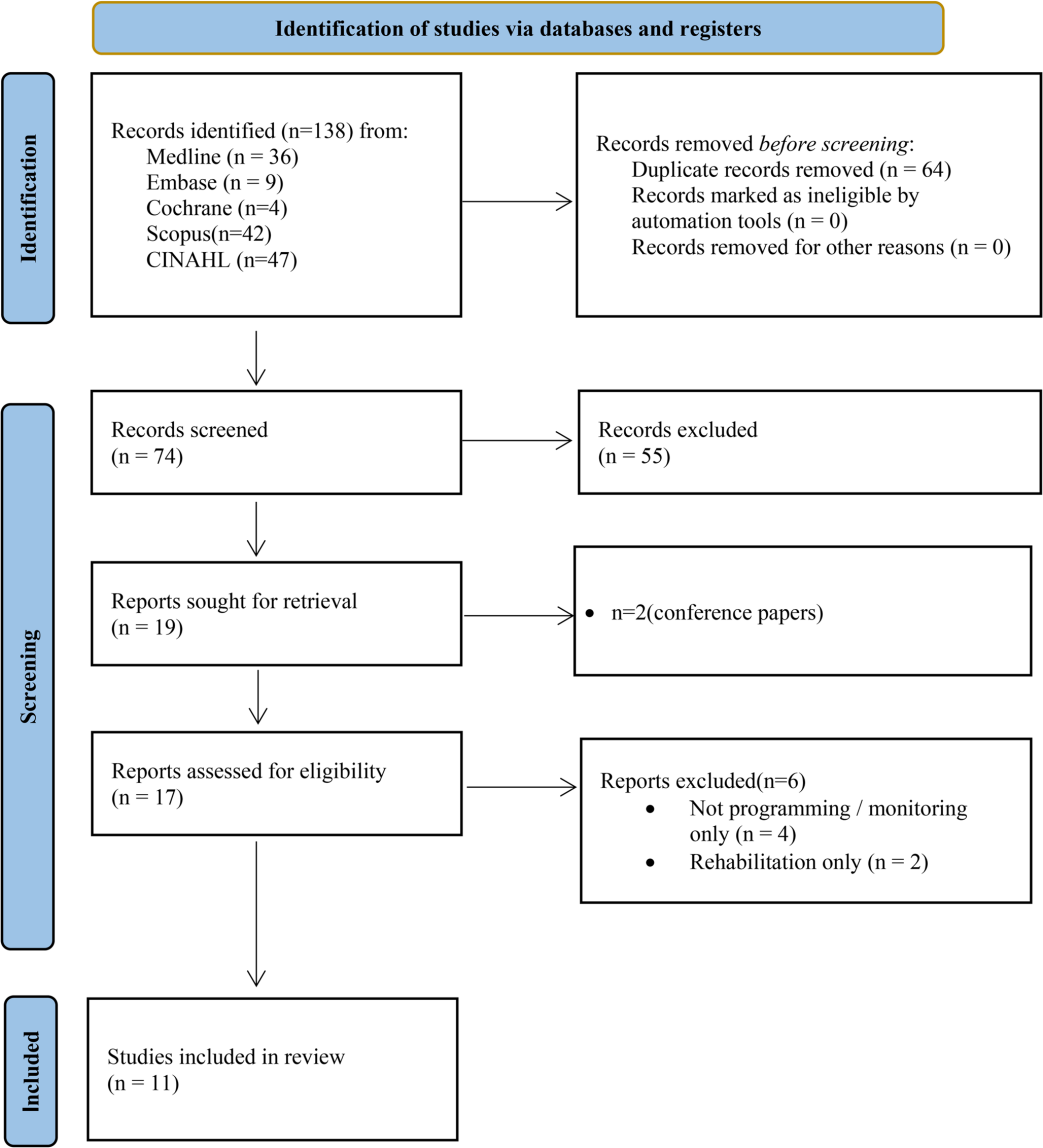
PRISMA flowchart for study identification and selection

Two independent reviewers screened titles, abstracts and full texts for eligibility. Reference lists of included articles were manually searched to identify additional studies. Eligible studies were those published in English that reported clinical outcomes (such as speech perception, aided thresholds, impedances or threshold/comfort levels) and/or implementation outcomes (such as feasibility, satisfaction or technical performance) in the context of remote CI programming. Review articles, conference abstracts, editorials and studies without original human data were excluded. Data were extracted on study design, participant characteristics, type of remote intervention, setting, devices or platforms used and reported clinical and implementation outcomes. Disagreements were resolved through discussion between reviewers. Risk of bias was assessed using the Cochrane Risk of Bias 2.0 tool for randomised trials [12] and the National Institutes of Health Quality Assessment Tool for observational studies [13]. Owing to heterogeneity in study designs and outcome measures, results were synthesised narratively (Fig. 1).

## Results

A total of 11 studies met the inclusion criteria, comprising 356 CI recipients (Table 1). Sample sizes ranged from 6 to 72 participants. Most participants were adults (*n* = 309; 86.7%), with 47 children and adolescents included across three mixed-age cohorts. The majority of studies recruited experienced CI users, although three studies included newly implanted individuals. Geographically, studies originated from Europe (*n* = 5), North America (*n* = 5), and South America (*n* = 1), with several multicentre collaborations reflecting variation in telehealth infrastructure. Study designs included one RCT, six prospective cohort or feasibility studies, two retrospective cohorts, one implementation study and one prospective cross-sectional comparative trial.

**Table 1:** Characteristics of included studies

Author/Year	Country	Study Design	Sample (A/C)	Mean Age & Range(years)	CI Experience (New/Experienced)	Model	Settings (clinic/home	Facilitator (Y/*N*)	Device / Platform	Follow-up Duration	Comparator	Primary Outcomes	Feasibility (session completion, %)	Satisfaction (instrument, score)	Technical issues	Industry involvement
Wesarg et al., 2010	Germany	RCT	57 (A) + 13(c)	35.6 (1–72)	Experienced	Synchronous	Clinic-to-clinic	Y	Cochlear Nucleus Remote Fitting	Two sessions	Y	T/C levels, mapping time, subjective feedback	98.6	Positive narrative	NR	Yes
Hughes et al., 2012	USA	Prospective ABA	29(A & C))	46(11–87)	Experienced (28) New user (1)	Synchronous	Clinic-to-clinic	Y	Telehealth video link, Cochlear Nucleus software	Three sessions	Y	Speech perception, ECAPs, thresholds	100	Positive	No technical issues	NR
Schepers et al., 2019	Germany	Prospective cohort	21(A) + 25(C)	C = 9.1(3.2–14.7) A = 57.6(25.5–80.1)	Experienced	Synchronous	Clinic-to-clinic	Y	MED-EL clinical platform	Two Sessions	Y	IFT, THR, MCL, Audiometry	100	High	No technical issues	Yes
Luryi et al., 2020	USA	Retrospective cohort	20(A)	A = 77.1(NR)	Mixed	Synchronous	Clinic-to-clinic	Y	Telemedicine CI mapping (AB, Cochlear, MED-EL)	Variable follow-up	Y	T/C, impedances, AzBio, IOI-CI	100	IOI-CI Positive	2 brief connection losses, no adverse events	NR
Meeuws et al., 2020	Belgium	Prospective feasibility	06(A)	A = 56.17(39–72)	New	Asynchronous(AI)	Simulated home	Y	FOX AI platform, Cochlear Nucleus software	Single session	N	Audiqueen self-tests + FOX AI (audiometry, phoneme, loudness)	100	High confidence & ease; high overall satisfaction.	2 pod faults, 1 connection drop	Yes
Nassiri et al., 2022	USA	Implementation study	A = NR	NR	Newly implanted	Hybrid	Clinic + remote	Y	Complete CI Care	12 months	N	CNC, AzBio, aided audiometry; CIQoL, Nijmegen CIQ.	data collection ongoing.	On going	NR	Yes
Ng et al., 2024	Canada	Prospective trial	39 (A)	A = 58(25–84)	Experienced	Synchronous	Home vs. host-site	Y	Zoom platform with-AB, Cochlear, MED-EL	Single session	Y	Aided thresholds, CNCAzBio	100	High (84–95%)	4 equipment mismatches + 1 audio issue no session failures	Yes
Lassaletta et al., 2025	Spain/Italy	Prospective	48 (A)	NR	Experienced	Synchronous	Clinic	Y	MED-EL e-assistant	Single session	N	bisyllable test, Free field	100	High(08/10 ratings)	Minor interruptions (≤ 20%); 2 sessions paused	Yes
Maruthurkkara et al., 2025	UK multicentre	Prospective & real-world	15(A)= (Clinical study)& 53(A) + 4(C)in real-world evaluation	63(	Experienced	Synchronous	Home	N	Cochlear remote Assist	Clinical=Single Real-world = ≤ 6 months	Y	CUNY sentences, system latency, task completion %, TUQ usability score	86.6%	High (5.8/7)	Latency ~ 1.7 s, no major issues	Yes
Samuel-Sierra et al., 2025	Brazil	Prospective cross-sectional	09(A)	46 (22–59)	Experienced	Asynchronous	Home	N	Remote Assistant Fitting (RAF)	2 Weeks	Y	T/C levels, Aided thresholds	100	High(09/10)	2 minor pairing issues; self-resolved; no session failures	Yes
Morton et al., 2025	USA	Prospective within-subject clinical trial	17 (12 A) + 5 C)	43(13–80)	Experienced	Synchronous (smartphone-based)	Home Vs Clinic	N	Advanced Bionics Remote Fitting App (smartphone)	Single remote + in-person visit	Y	AzBio sentences in quiet, impedances; fitting time	100%	High	Minor connection lags	Yes

*A *adults; *C *children; *RCT*  randomised controlled trial; *ABA *alternating baseline–treatment–baseline design; *CI*  cochlear implant; *MCL *most comfortable level; *THR *threshold level; *IFT *impedance field telemetry threshold; *T/C*  threshold and comfort levels;* IOI-CI* international outcome inventory for cochlear implant users; *CNC *consonant–nucleus–consonant word test; *AzBio *AzBio sentence test; *ECAP * electrically evoked compound action potential; *FOX AI* fitting to outcomes eXpert artificial intelligence platform; *RAF*  remote assistant fitting; *CIQoL * Cochlear implant quality of life instrument; *CIQ *Nijmegen cochlear implant questionnaire; *TUQ*  telehealth usability questionnaire; *NR *not reported; *Y/N*  Yes/No

Feasibility refers to session-completion rate; T/C levels denote the programming thresholds and comfort levels measured during cochlear-implant mapping

All studies evaluated remote CI programming and reported at least one clinical or implementation outcome. Three models of remote CI programming were described. Eight studies evaluated synchronous programming involving real-time clinician access to programming software. Two studies investigated asynchronous or semi-automated systems, including the FOX artificial-intelligence platform and remote assistant fitting. One study implemented a hybrid model combining in-clinic and remote follow-up. Nine studies reported quantitative clinical outcomes. Eight assessed speech perception using CNC words, AzBio or BKB sentences, or digits-in-noise measures. In all six comparator studies, speech perception outcomes following remote programming were equivalent to those obtained with in-person programming, with no evidence of deterioration. One asynchronous feasibility study also demonstrated comparable outcomes. Five studies reported aided thresholds, which remained stable across programming modes. Five studies evaluated impedances and T/C stimulation levels, showing no clinically meaningful changes in device parameters after remote sessions. Evidence from three mixed-age cohorts indicated that paediatric recipients achieved programming stability comparable to adults, although sample sizes were small. All eleven studies reported implementation-related findings (Table 2).

**Table 2:** Summary of clinical and implementation outcomes

Author (Year)	Model	Clinical Equivalence	Key Quantitative Outcomes	Implementation Insights	Technical Reliability
Wesarg et al., 2010	Synchronous	✓	Stable T/C levels; aided thresholds unchanged	High satisfaction (≥ 90%)	Minor connection issues
Hughes et al., 2012	Synchronous	✓	CNC, ECAP comparable; speech *p* > 0.05	100% completion; mean duration 131–138 min	Latency <5s
Schepers et al., 2019	Synchronous	✓	MCL/THR stable; speech unchanged	Feasible across ages; caregiver crucial	Occasional delays
Luryi et al., 2020	Synchronous	✓	AzBio, impedances; comfort levels stable	High satisfaction (IOI-CI > 80%)	2 disconnections; resolved
Meeuws et al., 2020	Asynchronous (AI/FOX)	≈	T/C stable; self-test validated	100% completion; comfort high	2 wireless-pod failures; no session loss
Nassiri et al., 2022	Hybrid	✓	Speech & thresholds comparable; ongoing cohort	Feasible integrated CI care model	Prep time burden for staff
Ng et al., 2024	Synchronous	✓	Equivalent outcomes between laptop vs. hosted sites	Usability 84–95%; 100% session completion	Minor setup issues
Lassaletta et al., 2025	Synchronous	✓	Speech (Italian bisyllabic test)	100% sessions completed; user/clinician acceptance high	Minor interruptions (< 20%)
Maruthurkkara et al., 2025	Synchronous (App)	✓	CUNY sentences; TUQ usability	86% completion; high usability; latency ~ 1.7s	No failures
Samuel-Sierra et al., 2025	Asynchronous (RAF)	✓	T/C stability; aided thresholds	100% completion; mean satisfaction 8–9/10	2 minor pairing issues; self-resolved
Morton et al., 2025	Synchronous (smartphone-based remote programming)	✓	AzBio, impedances, Fitting time	100% completion; user/clinician acceptance high	Brief connection lag; no failures.

*MCL *most comfortable level; *THR *Threshold Level; *IOI-CI *international outcome inventory for cochlear implant users; *CNC *consonant–nucleus–consonant test; *AzBio * AzBio sentence test; *ECAP * electrically evoked compound action potential; *FOX AI *fitting to outcomes eXpert AI platform; *RAF *remote assistant fitting; *TUQ *telehealth usability questionnaire

CI recipient satisfaction was consistently high, with reduced travel burden, convenience, and scheduling flexibility frequently cited. Clinician satisfaction was also positive, although some platforms required additional preparation or troubleshooting. Technical challenges, typically unstable internet connections or software interruptions were common but were usually resolved without affecting clinical outcomes. Studies involving paediatric participants highlighted the importance of caregiver involvement, while several studies identified limited digital literacy as a barrier, particularly among older adults. Eight of the eleven studies included in-person programming as a comparator arm. Across all comparator studies, clinical outcomes were equivalent between remote and standard programming. Several studies also reported practical advantages of remote models, including high session-completion rates and usability across different clinical environments.

Risk-of-bias assessments are summarised in Table 3. The single randomised controlled trial was judged low risk using the Cochrane RoB 2.0 tool. The remaining ten observational studies were rated as fair to good quality, reflecting limitations relating to sampling, non-randomised design, and incomplete control of confounding. A prospective comparative trial and a smartphone-based clinical trial were rated as good quality owing to structured methodology and transparent reporting. Most studies relied on objective measures reducing the likelihood of measurement bias.

**Table 3:** Risk of bias and quality assessment of included studies

Study	Design	Assessment Tool	Randomization / Selection	Deviations / Confounding	Missing Data / Attrition	Outcome Measurement Validity	Selective Reporting	Overall Risk of Bias / Quality Rating
Wesarg et al., 2010	Randomized Controlled Trial	Cochrane RoB 2.0	Low risk	Low risk	Low risk	Low risk	Low risk	Low risk
Hughes et al., 2012	Prospective feasibility study (non-randomized)	NIH	Fair	Fair	Low risk	Low risk	Low risk	Fair
Schepers et al., 2019	Prospective cohort study	NIH	Fair	Fair	Low risk	Low risk	Low risk	Fair
Luryi et al., 2020	Retrospective cohort study	NIH	Fair	Fair	Low risk	Low risk	Low risk	Fair
Meeuws et al., 2020	Prospective feasibility study	NIH	Fair	Fair	Low risk	Low risk	Low risk	Fair
Nassiri et al., 2022	Hybrid (implementation + feasibility)	NIH	Fair	Fair	Low risk	Low risk	Low risk	Fair
Ng et al., 2024	Prospective trial (non-randomized)	NIH	Good	Fair	Low risk	Low risk	Low risk	Good
Lassaletta et al., 2025	Prospective multicentre pilot study	NIH	Fair	Fair	Low risk	Low risk	Low risk	Fair
Maruthurkkara et al., 2025	Prospective real-world cohort study	NIH	Fair	Fair	Low risk	Low risk	Low risk	Fair
Samuel-Sierra et al., 2025	Prospective cross-sectional study	NIH	Fair	Fair	Low risk	Low risk	Low risk	Fair
Morton et al., 2025	Prospective within-subject clinical trial	NIH	Good	Low risk	Low risk	Low risk	Low risk	Good

*NIH *national institutes of health quality assessment tool

## Discussion

This systematic review synthesised evidence from eleven studies and demonstrates that remote CI programming is clinically viable and broadly acceptable across synchronous, asynchronous and hybrid delivery models. Across the available evidence, clinical outcomes, including speech perception, aided thresholds and device integrity were comparable to in-person programming, and satisfaction among CI recipients and clinicians was consistently high. At the same time, several challenges emerged, particularly digital literacy barriers among older adults, connectivity limitations and the need for caregiver support in paediatric settings.

Clinical outcomes across the included studies consistently indicated stability during remote programming. Speech perception measured using CNC, AzBio, BKB or digits-in-noise tests showed no deterioration following remote sessions [14–16], and aided thresholds, impedances and T/C levels were similarly unchanged [16, 17]. These findings were evident across synchronous, asynchronous and hybrid models [18–20], suggesting that stable programming outcomes are achievable across different telehealth configurations. Limited paediatric data also suggested stable programming behaviour when caregivers were actively involved [16, 21]. These results extend earlier work, including Shah et al. [22], and are consistent with broader tele-audiology evidence demonstrating comparable outcomes between remote and in-person care [23, 24]. More recent multicentre work has further strengthened confidence in the safety and feasibility of remote CI programming [25].

User experience was a consistent strength across the studies. CI recipients frequently reported reduced travel burden, improved convenience and greater flexibility [16, 25], while caregivers in paediatric-inclusive studies described remote participation as empowering [18]. Clinicians generally found remote CI programming feasible and effective, though some workflows, especially asynchronous or patient-assisted models, required additional preparation or troubleshooting [19, 20, 26]. These findings agree with wider telehealth literature, where convenience and reduced logistical burden are major contributors to satisfaction [23, 24]. However, most studies relied on informal feedback rather than validated instruments and structured assessment of clinician workload was rare. Future evaluations should incorporate standardised patient-reported outcomes and clear indicators of clinician time and effort.

Technical reliability played an important role in shaping feasibility. Several studies reported intermittent connectivity issues or temporary software interruptions during synchronous sessions, although these did not affect clinical outcomes [15, 16, 20, 27]. Asynchronous or patient-assisted systems were less dependent on bandwidth but required reliable hardware and accurate user-generated data [20, 26]. Reporting of embedded safety safeguards such as telemetry limits, rollback functions or automated logs was limited [15, 18], making it difficult to fully evaluate system-level safety. Encouragingly, smartphone-based systems showed stable connectivity and no device-related adverse events [25]. Broader determinants of digital access including socioeconomic factors, language support, reimbursement structures and home-internet variability were generally under reported despite their recognised importance in digital health [28, 29]. The lack of cost-effectiveness analysis further restricts understanding of system-level feasibility.

Paediatric evidence remains limited and must be interpreted cautiously. Only 42 children were represented across two mixed-age cohorts [16, 21], and successful remote CI programming depended heavily on caregiver facilitation for setup, environmental control and behavioural responses. Although clinical stability was reported, the small sample sizes and absence of child-reported or caregiver-burden outcomes limit conclusions. This aligns with wider paediatric telehealth literature in which parental involvement can both support and constrain participation [30]. At present, remote CI programming should be considered a supplementary follow-up option in children rather than a replacement for in-person care.

## Limitations

This review has several limitations. Most included studies were small observational cohorts with modest methodological quality, and only one randomised trial was available. Paediatric representation was minimal, outcome measures varied across studies and technical reporting was inconsistent. No studies examined cost-effectiveness or long-term outcomes of sustained remote follow-up. Future research should prioritise multicentre comparative trials, paediatric-specific evaluations, assessment of caregiver burden, economic analyses and rigorous testing of emerging AI-assisted and patient-adjusted approaches with clear safety criteria.

## Conclusion

Remote CI programming is feasible, clinically stable and well accepted by CI recipients and clinicians. However, limited paediatric data, inconsistent technical reporting and the absence of economic evaluations constrain confidence in large-scale implementation. Progressing from a promising adjunct to a fully validated component of CI follow-up care will require standardised technical reporting, robust comparative studies and deliberate strategies to ensure equitable access across diverse user groups.

## Data Availability

Data sharing not applicable to this article as no datasets were generated or analysed during the current study.
